# Association of furosemide versus chlorothiazide exposures with serum sodium, potassium, and chloride among infants with bronchopulmonary dysplasia

**DOI:** 10.1038/s41372-024-02159-z

**Published:** 2024-11-05

**Authors:** Timothy D. Nelin, Matthew Huber, Erik A. Jensen, Sara B. DeMauro, Heidi Morris, Scott A. Lorch, Kathleen Gibbs, Stamatia Alexiou, Natalie Napolitano, Anna Bustin, Nicolas A. Bamat

**Affiliations:** 1 Division of Neonatology, Department of Pediatrics, Children’s Hospital of Philadelphia, Philadelphia, PA USA; 2 Department of Pediatrics, University of Pennsylvania Perelman School of Medicine, Philadelphia, PA USA; 3 Center of Excellence in Environmental Toxicology, University of Pennsylvania Perelman School of Medicine, Philadelphia, PA USA; 4 Leonard Davis Institute of Health Economics, Philadelphia, PA US; 5 Newborn/Infant Chronic Lung Disease Program, Children’s Hospital of Philadelphia, Philadelphia, PA US; 6 Division of Pulmonary Medicine, Department of Pediatrics, Children’s Hospital of Philadelphia, Philadelphia, PA USA; 7 Respiratory Therapy Department, Children’s Hospital of Philadelphia, Philadelphia, PA US; 8 Department of Pharmacy Services, Children’s Hospital of Philadelphia, Philadelphia, PA USA

**Keywords:** Adverse effects, Respiratory tract diseases

## Abstract

**Objectives:**

To examine the association of novel furosemide versus thiazide diuretic exposure with changes in serum sodium, potassium, and chloride levels among infants with grade 2/3 bronchopulmonary dysplasia (BPD).

**Study Design:**

Retrospective cohort study of infants admitted to a level IV neonatal intensive care unit (NICU) with grade 2/3 BPD. We measured within-subject change in serum sodium, potassium, and chloride before and after diuretic initiation using multivariable regression to adjust for differences in dosing and clinical covariates.

**Results:**

We identified 94 infants contributing 137 novel diuretic exposures. No significant difference was noted in the association between chlorothiazide versus furosemide and serum sodium, potassium, or chloride change in multivariable modeling.

**Conclusions:**

Changes in serum electrolytes were similar for chlorothiazide and furosemide, questioning the perception that chlorothiazide leads to less electrolyte derangement among preterm infants with grade 2/3 BPD.

## Introduction

Bronchopulmonary dysplasia (BPD) is the most common chronic morbidity of preterm birth [1–3]. Identifying safe and effective therapeutic interventions for infants with high-grade BPD is vital to improving outcomes. Infants with high-grade BPD are exposed to numerous medications throughout hospital admission. Diuretics are the most used medication class, with furosemide and chlorothiazide being the most frequently prescribed medications despite limited efficacy and safety data for these drugs [4–8]. Although they are known to affect electrolyte levels in adults and children [9–12], data comparing the impact of furosemide and chlorothiazide on plasma electrolyte levels in infants with high-grade BPD are lacking.

Randomized controlled trials and observational studies have demonstrated an association between diuretic use and electrolyte derangements in infants with BPD receiving chronic diuretic therapy [13–17]. Clinical outcomes related to diuretic use in infants with high-grade BPD and the impact of subsequent electrolyte derangements on outcomes remain largely unknown. In the absence of evidence, a wide variation in practice exists among providers and hospitals that prescribe furosemide and chlorothiazide [7, 8, 18]. Furosemide, a loop diuretic, inhibits electrolyte reabsorption from the renal lumen in the thick ascending limb of the loop of Henle and has a greater maximal diuretic effect [19]. Chlorothiazide works on the distal convoluted tubule. As less sodium resorption occurs in the distal convoluted tubule compared to the loop of Henle, chlorothiazide has a weaker maximal diuretic effect. This weaker diuretic effect is believed to be associated with lesser electrolyte derangements for chlorothiazide than furosemide in clinical practice [7, 17, 19, 20]. Our recent work questions this presumption. We found that infants with chronic thiazide diuretic exposure were more likely to receive sodium chloride (NaCl) and potassium chloride (KCl) supplementation than infants with chronic loop diuretic exposure [21]. However, we did not measure plasma electrolyte levels directly, rather we used NaCl and KCl supplementation as an indicator of clinically meaningful electrolyte wasting.

To address this gap in knowledge, we assessed the association between novel exposures to furosemide and chlorothiazide and subsequent change in serum sodium, potassium, and chloride levels. We hypothesized that the decrease in serum sodium, potassium, and chloride levels would be more pronounced after exposure to furosemide compared to chlorothiazide in infants with high-grade BPD.

## Methods

### Study population

We performed a retrospective cohort study in a convenience sample of infants born between October 1, 2010 and March 31, 2020 who were admitted to the Children’s Hospital of Philadelphia. Our study population included infants diagnosed with grade 2/3 BPD with a postmenstrual age (PMA) of 36–60 weeks. We used the 2019 NRN Criteria for grade 2 and 3 BPD, defined as the use of >2 L/min of nasal cannula, non-invasive positive airway pressure, or invasive mechanical ventilation at 36 weeks’ PMA among infants born < 32 weeks’ GA [22]. Infants were excluded if they had short gut syndrome or a congenital anomaly that plausibly impacted renal function. Diuretic exposures were excluded if they occurred while the infant was on extracorporeal membrane oxygenation (ECMO). Data collection was approved by the Institutional Review Board of the Children’s Hospital of Philadelphia (IRB #19-016420).

### Exposures and outcomes

**Fig. 1:**
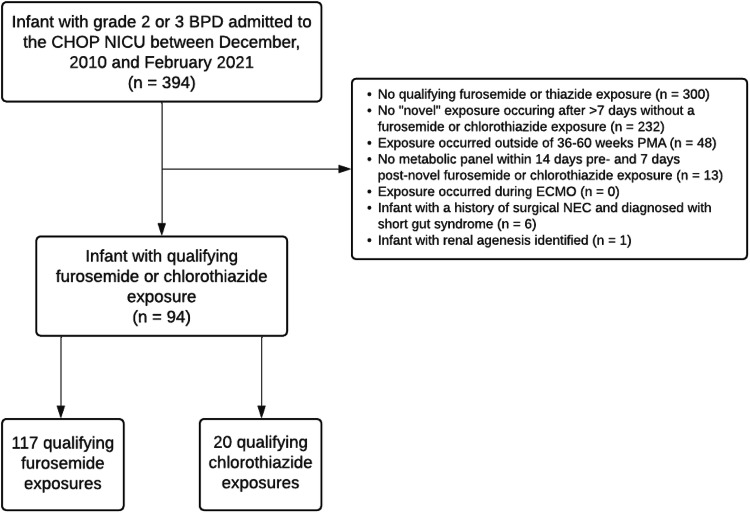
Cohort Creation Flowchart. Flowchart depicts identification of study cohort of very preterm infants with grade 2/3 BPD with a qualifying furosemide or chlorothiazide exposure between 36 and 60 weeks’ PMA.

Novel treatment with furosemide or chlorothiazide was our primary exposure of interest. Our institution does not have a clinical practice guideline for diuretic use in BPD and exposures reflect individual provider preferences. We defined novel diuretic exposure as the administration of at least one dose of diuretic to infants who had not received any diuretic in the preceding 7 days and had a baseline serum electrolyte panel drawn within 14-days before the diuretic exposure to provide baseline electrolyte values and a follow-up electrolyte panel drawn within 7 days after the diuretic exposure (Fig. 1). We chose a 7-day period without receiving any diuretic to minimize potential carry-over effects from prior administrations. Enteral administrations were converted to IV equivalents to standardize dose exposure using a bioavailability of 50% for furosemide and 20% for chlorothiazide [19].

Absolute changes in serum sodium, potassium, and chloride concentration after novel diuretic exposure were our primary outcomes. We measured these changes by comparing sodium, potassium, and chloride levels obtained from metabolic panels 12 h to 7 days after novel diuretic exposure with baseline electrolyte levels. We chose the range of 12 h to 7 days to account for variability among clinical providers in when repeat serum electrolyte levels are obtained after diuretic administration. For infants with multiple electrolyte panels drawn within the baseline and follow-up periods, we selected the baseline and follow-up panel closest in time to the diuretic exposure.

### Statistical analysis

We summarized cohort characteristics using standard descriptive statistics. We used multivariable regression to measure within-subject change in serum sodium, potassium, and chloride before and after diuretic initiation. Post-menstrual age (PMA) at exposure, the amount of time elapsed between the diuretic exposure and the post-treatment electrolyte measurement, the number of diuretic doses received from initiation until follow-up laboratory assessment, and low (≤ 1 mg/kg/24 h for furosemide and ≤ 10 mg/kg/24 h for chlorothiazide) vs high (> 1 mg/kg/24 h for furosemide and > 10 mg/kg/24 h for chlorothiazide) diuretic dose were included as covariates a priori. Additional candidate covariates included: diuretic route of administration, sex, race, ethnicity, baseline TPN use, baseline enteral NaCl and KCl supplementation, increase in enteral or parenteral NaCl or KCl between baseline and post-exposure electrolyte measures, and concurrent hydrocortisone, dexamethasone or dopamine use. We considered dopamine specifically as it is the most common vasoactive medication in this population at our institution and because it has known effects on renal blood flow [23]. For each electrolyte, we performed a bivariable regression of candidate covariates with within-subject changes in sodium, potassium, and chloride. Candidate covariates were included in multivariable regression if they were associated with electrolyte level changes at a significance of *p* < 0.10 in bivariable analysis. Increase in NaCl supplementation met inclusion criteria for the multivariable model for sodium. Concurrent dexamethasone use and infant sex met inclusion for the multivariable model for chloride. Diuretic route of administration met inclusion for the multivariable model for potassium. We used cluster-robust variance estimates to account for repeated observations within subjects. To allow for clinically relevant reporting, we estimated the changes in serum sodium, potassium, and chloride using post-estimation margins [24]. A two-sided *p-*value of < 0.05 indicated statistical significance. All statistical analyses were performed with Stata 18 (StataCorp, College Station, Texas, USA).

## Results

Table 1 displays cohort characteristics. We identified 94 infants with grade 2/3 BPD. Among these 94 infants, we identified 137 novel diuretic exposures, including 20 novel chlorothiazide exposures and 117 novel furosemide exposures. Table 2 displays clinical characteristics of the novel chlorothiazide and furosemide exposures.

**Table 1:** Cohort characteristics (*n* = 94).

***Baseline Characteristics***	
Gestational age, median [IQR], week	25 [23–27]
Birth weight, median [IQR], grams	658 [485–831]
Maternal ethnicity, No. (%)	
Not Hispanic or Latino	78 (83)
Hispanic or Latino	16 (17)
Maternal race, No. (%)	
White	17 (18)
Black	42 (45)
Other or unknown	35 (37)
Sex, No. (%)	
Female	38 (40)
Male	56 (60)
***Clinical Characteristics***	
Respiratory support at 36 weeks PMA, No (%)	
Non-invasive CPAP	24 (25)
Non-invasive bi-level support	1 (1)
Conventional mechanical ventilation	62 (66)
High frequency ventilation	7 (8)
Novel diuretic exposures^a^, No. (%)	
Furosemide	117 (85)
Chlorothiazide	20 (15)
Novel diuretic exposures per infant, No. (%)	
1 exposure	63 (67)
2 exposures	25 (27)
≥ 3 exposures	6 (6)

*IQR* interquartile range, *PMA* post-menstrual age, *CPAP* continuous positive airway pressure.

^a^Accounts for total number of novel diuretic exposures within the cohort. Total number of exposures is 137, which exceeds 94 subjects as each subject could contribute multiple exposures if they met criteria.

**Table 2:** Characteristics of novel chlorothiazide and furosemide exposures (*n* = 137).

Characteristics	Chlorothiazide	Furosemide	*p*-value
Respiratory support at diuretic exposure, No. (%)			0.24
Non-invasive CPAP	6 (30)	27 (23)	
Non-invasive bi-level support	1 (5)	1 (1)	
Conventional mechanical ventilation	13 (65)	78 (67)	
High frequency ventilation	0 (0)	11 (9)	
PMA at diuretic exposure (weeks), mean (SD)	46 (7)	44 (7)	0.16
High diuretic dose exposure, No. (%)	6 (30)	35 (30)	0.99
Route of diuretic exposure, No. (%)			< 0.001
Oral, nasogastric, or gastrostomy tube	14 (70)	24 (20)	
Nasoduodenal	4 (20)	17 (15)	
Intravenous	2 (10)	76 (65)	
Doses between initiation and post-exposure lab, No. (%)			< 0.001
1	0 (0)	61 (52)	
2	5 (25)	31 (27)	
3	2 (10)	8 (7)	
4 or greater	13 (65)	17 (14)	
Exposure to TPN during exposure, No. (%)	4 (20)	52 (44)	0.04
Enteral NaCl supplementation at exposure, No. (%)	1 (5)	11 (9)	0.52
Enteral KCl supplementation at exposure, No. (%)	0 (0)	4 (3)	0.40
Na supplementation increase during exposure, No. (%)	0 (0)	8 (7)	0.23
K supplementation increase during exposure, No. (%)	1 (5)	14 (12)	0.36
Cl supplementation increase during exposure, No. (%)	1 (5)	17 (14)	0.24
Exposure and follow-up lab time difference (hours), mean (SD)	43.8 (22.8)	54.4(42.0)	0.28
Baseline and follow-up lab time difference (hours), mean (SD)	156 (83)	115 (86)	0.05
Hydrocortisone during exposure, No. (%)	0 (0)	28 (24)	0.01
Dexamethasone during exposure, No. (%)	2 (10)	5 (4)	0.28
Dopamine during exposure, No. (%)	0 (0)	3 (3)	0.47

*CPAP* continuous positive airway pressure, *PMA* post-menstrual age, *TPN* total parenteral nutrition, *NaCl* Sodium chloride, *KCl* Potassium chloride, *Na* sodium, *K* Potassium, *Cl* Chloride, *SD* standard deviation

**Fig. 2:**
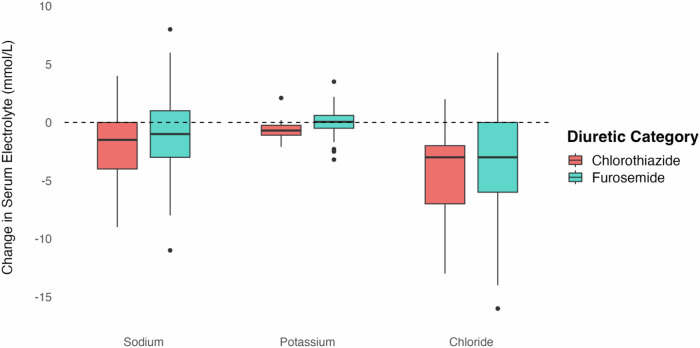
Change in serum electrolyte after novel diuretic exposure. Figure depicts the unadjusted change in serum sodium, potassium, and chloride after novel chlorothiazide and furosemide exposure. No significant difference was noted between furosemide and chlorothiazide with regard to change in serum sodium (−0.9 vs −1.9 mmol/L, *p* = 0.25) or chloride (−3.1 vs −4.5 mmol/L, *p* = 0.19). Compared to novel furosemide, novel chlorothiazide was associated with greater change in serum potassium (−0.0 vs −0.6, *p* = 0.008).

Figure 2 displays the unadjusted changes in serum sodium, potassium, and chloride after furosemide vs chlorothiazide exposure. There was no significant difference in serum sodium or chloride change with furosemide vs chlorothiazide administration, but there was a significant decrease in serum potassium (0.0 vs −0.6 mmol/L, *p* = 0.008). The results of multivariable logistic regression modeling for changes in serum electrolyte following novel furosemide vs novel chlorothiazide administration are displayed in Table 3. After covariate adjustment, there was no significant difference in the change in serum sodium (−1.1 mmol/L vs −0.9 mmol/L, *p* = 0.813), potassium (0.0 vs −0.7 mmol/L, *p* = 0.080), or chloride (−3.4 vs −2.8 mmol/L, *p* = 0.567) following novel furosemide vs chlorothiazide administration (Table 3).

**Table 3:** Adjusted change in serum sodium, potassium, and chloride levels following novel furosemide or chlorothiazide administration.

	Change in serum Na, marginal means (mmol/L)	*p-*value	Change in serum K, marginal means (mmol/L)	*p-*value	Change in serum Cl, marginal means (mmol/L)	*p*-value
*Novel Exposure*						
Furosemide	−1.1	Ref.	0.0	Ref.	−3.4	Ref.
Chlorothiazide	−0.9	0.813	−0.7	0.080	−2.8	0.567
*PMA at Exposure*						
36–40 weeks	−1.2	Ref.	0.0	Ref.	−3.6	Ref.
40–44 weeks	−1.5	0.511	−0.1	0.663	−3.5	0.858
44–48 weeks	−1.1	0.888	−0.5	0.107	−3.7	0.924
>48 weeks	−0.6	0.431	0.0	0.829	−2.5	0.148
Diuretic exposure to follow-up lab time difference^a^	0.02	0.003	0.003	0.085	0.04	< 0.001
Diuretic doses between initiation and follow-up lab time^a^	−0.443	0.026	0.049	0.360	−0.746	0.004
*Diuretic Dose*^b^						
Low	−0.5	Ref.	0.0	Ref.	−2.7	Ref.
High	−2.5	< 0.001	−0.4	0.285	−4.6	0.021
**Electrolyte-Specific Covariates**						
No Increase in NaCl	−1.1	Ref.				
Increase in NaCl^c^	−0.3	0.418				
*Diuretic Route*						
Intravenous			−0.1	Ref.		
Gastric			−0.2	0.932		
Post-Pyloric			−0.2	0.505		
No Dexamethasone					−3.2	Ref.
Dexamethasone					−5.2	0.306
Male Sex					−2.8	Ref.
Female Sex					−3.6	0.290

*Na* Sodium, *K* Potassium, *Cl* Chloride.

Table depicts marginal means for change in serum Na, K, and Cl between pre- and post-lab values before and after novel diuretic administration estimated with a multivariable regression model using cluster-robust variance estimates to account for repeated observations within subjects. PMA at exposure, diuretic exposure to follow-up lab time difference, and dose were included a priori. Increase in NaCl supplementation during the exposure window between the pre- and post-lab times for sodium, concurrent dexamethasone use and sex for chloride, and medication route for potassium were included following association with novel furosemide and chlorothiazide exposure at *p* < 0.10 in bivariable analysis.

^a^Continuous variable displayed as beta-coefficient instead of marginal mean.

^b^Dose for furosemide and chlorothiazide, low denotes ≤1 mg/kg/24h for furosemide and ≤10 mg/kg/24h for chlorothiazide, high denotes >1 mg/kg/24h for furosemide and >10 mg/kg/24h for chlorothiazide.

^c^Denotes increase in enteral or parenteral NaCl supplementation between diuretic exposure and post-lab collection.

A higher (>1 mg/kg/24 h for furosemide and >10 mg/kg/24 h for chlorothiazide) compared to lower (≤1 mg/kg/24 h for furosemide and ≤10 mg/kg/24 h for chlorothiazide) diuretic dose was associated with greater serum sodium (−2.5 vs −0.5 mmol/L, *p* < 0.001) and chloride loss (−4.6 vs −2.7 mmol/L, *p* = 0.021), but not potassium loss (−0.4 vs 0.0 mmol/L, *p* = 0.285). Exposure to more doses of diuretic between novel diuretic initiation and follow-up metabolic panel collection was associated with greater sodium and chloride loss, but not potassium loss (Table 3). A longer time interval between novel diuretic initiation and follow-up metabolic panel collection was associated with a smaller change in serum sodium, potassium, and chloride (Table 3).

## Discussion

In a contemporary cohort of preterm infants with grade 2 or 3 BPD, we did not find a significant difference in serum sodium, potassium, or chloride changes following administration of furosemide compared to chlorothiazide. These findings suggest that chlorothiazide may not be a “gentler” diuretic than furosemide when characterized based on serum sodium, potassium, and chloride loss in preterm infants with high grade BPD.

Our previous findings also call into question whether chlorothiazide has less pronounced effects on serum electrolytes. In a cohort of 3252 very preterm infants developing high-grade BPD at United States children’s hospitals, we showed that chronic thiazide use was associated with greater sodium chloride and potassium chloride supplementation compared to chronic loop diuretic exposure [21]. Our findings are inconsistent with a previous study by Dartois et al., who reported a lower risk of electrolyte derangement with thiazide diuretics compared to furosemide (37% vs. 87%, *p* < 0.001) exposure in infants born between 36 and 41 weeks’ gestational age. However, this study evaluated a broad neonatal population that included preterm and term infants, and no significant difference in electrolyte derangements were noted among the preterm infant population born < 32 weeks’ gestation [17]. The differing findings between our studies may reflect heterogenous treatment effects in distinct neonatal populations.

We identified several clinical and diuretic characteristics that were associated with greater changes in serum electrolytes following furosemide or chlorothiazide administration. A higher diuretic dose and exposure to a higher number of diuretic administrations were associated with greater serum sodium and chloride loss. Our findings of greater electrolyte change following exposure to a higher diuretic dose and higher number of diuretic administrations are consistent with findings from two recent studies. Sridharan et al. found that a cumulative dose of 4 mg/kg/day of furosemide increased the risk of electrolyte abnormality among preterm infants in a single-center study [25]. Similarly, Dartois et al. found that a total daily dose of chlorothiazide of 15 mg/kg was associated with a higher risk of serum electrolyte disturbance or prescription of electrolyte supplementation compared to a total daily dose of 10 mg/kg [17]. Our findings add to this evidence demonstrating an association between higher diuretic exposure and greater change in serum sodium and chloride.

We found that as a greater amount of time elapsed between diuretic exposure and follow-up laboratory collection, there was a smaller change in serum sodium, potassium, and chloride. We speculate that this finding may reflect the influence of time and counterregulatory mechanisms in response to initial electrolyte loss over time. While we are not aware of any contemporary studies that report similar findings, recently published data report rapid tolerance to furosemide diuresis. In a retrospective cohort study of repeatedly dosed furosemide in a similar BPD population, there was a decrease in net fluid balance in the first 24 h following furosemide initiation, followed by a progressively diminishing diuretic effect over the next 48 h [26]. In the context of our study, qualifying exposures included the receipt of a single diuretic dose or the first dose of a prolonged course of diuretic. Electrolyte values collected after a longer duration from diuretic initiation may reflect the attenuating impact of time and diuretic tolerance on electrolyte derangements.

As numerous physiologic processes contribute to sodium, potassium, and chloride homeostasis, it is plausible that alternate biological mechanisms associated with electrolyte homeostasis may have contributed to our null findings, including perturbation of the hypothalamic-pituitary-adrenal axis or acute kidney injury. Adrenal insufficiency is commonly observed in infants with BPD due to prematurity and steroid exposure during NICU admission [27]. We controlled for the receipt of hydrocortisone in our model, baseline electrolyte supplementation, and increases in electrolyte supplementation during the novel diuretic exposure period therefore we speculate that adrenal insufficiency and potentially associated electrolyte derangements were unlikely to contribute to our findings. While we did not assess baseline creatinine, eGFR, or urine output in our model to account for acute kidney injury, a strength of the study includes the pre-post study design. Our assessment of within-subject change in serum sodium, potassium, and chloride would attenuate confounding due to baseline differences in renal function between groups. Additionally, we defined novel exposures to furosemide and chlorothiazide conservatively, only including infants with no diuretic exposures in the preceding 7-days. Therefore, we speculate that differences in acute kidney injury are unlikely to have contributed to the null findings of the study.

Our study has several limitations. First, it is a retrospective cohort study relying on clinical data extracted from the electronic health record, allowing for the possibility of misclassification bias from inaccurate documentation. Second, we encountered variability in the timing of electrolyte measurements relative to diuretic exposures. To mitigate bias, we included the timing of electrolyte measurement and the cumulative number of diuretic administrations received in our multivariable modeling and reported the independent effect of this expected variation in clinical practice on the observed electrolyte levels. Heterogeneity in blood sampling method (i.e. capillary vs free-flowing venous sample vs arterial sample) may also affect the reported electrolyte values. While our cohort of 94 infants is the largest cohort study evaluating serum electrolyte derangements after diuretic exposures in preterm infants with high grade BPD, our findings reflect a single center experience, and may not generalize to alternative diuretic dosing strategies. Lastly, our comparison was limited to 20 novel chlorothiazide exposures, raising the possibility of a type 2 error. To explore this limitation, we considered power calculations using a traditional approach for two independent means, assuming an alpha of 0.05 with our observed sample size and variances. We considered a change of 4 mmol/L for sodium, 1 mmol/L for potassium, and 5 mmol/L for chloride as minimally important clinical differences. Based on the subjects and data we obtained, the estimated power to detect these differences were 99% for sodium, 98% for potassium, and 99% for chloride. Although these power estimates do not reflect the full complexity of our data structure and multivariable model, they provide pragmatic reassuring estimates that our sample was adequate to detect clinically relevant difference in electrolyte changes.

Our findings contribute data to the understudied area of diuretic therapy in preterm infants with BPD. In keeping with results from our previous study, we found that chlorothiazide is not associated with less electrolyte loss compared to furosemide, challenging the assumption that it is a “gentler” diuretic in this context. Clinicians may exercise caution when considering the preferential use of thiazide diuretics if the goal is to exert a diuretic effect while lessening the impact of sodium, potassium, and chloride loss. Future studies should investigate differences in additional side effects associated with diuretic administration including calcium losses and nephrolithiasis. This study highlights the need for future prospective studies that measure electrolyte levels in a standardized manner and randomize infants to furosemide vs chlorothiazide to properly compare treatment efficacy and safety in preterm infants with BPD.

## Data Availability

The datasets generated during/or analyzed during the current study and the code used to analyze and manage the data are available from the corresponding author on reasonable request.
